# A national system for monitoring intensive care unit demand and capacity: the Critical Health Resources Information System (CHRIS)

**DOI:** 10.5694/mja2.50988

**Published:** 2021-03-28

**Authors:** David Pilcher, Nicholas R Coatsworth, Melissa Rosenow, Jason McClure

**Affiliations:** ^1^ Alfred Health Melbourne VIC Australia; ^2^ Centre for Outcome and Resource Evaluation Australian and New Zealand Intensive Care Society Melbourne VIC; ^3^ Chief Medical Officer Group Australian Government Department of Health Canberra ACT; ^4^ Adult Retrieval Victoria Ambulance Victoria Melbourne VIC

**Keywords:** Intensive care, COVID‐19, Infectious diseases, Respiratory tract infections, Information management, Information storage and retrieval

CHRIS supported the Victorian ICU response during the COVID‐19 pandemic

The coronavirus disease 2019 (COVID‐19) pandemic put an unprecedented strain on intensive care resources throughout the world. Initially in Wuhan (China)^1^ and then in Lombardy (Italy),^2^ London (United Kingdom) and New York (United States),^3^ demand exceeded capacity, with 10–15% of the patients admitted to hospital developing critical illness. Australia has 191 adult and paediatric intensive care units (ICUs), with over 2300 ICU beds.^4^ This is equivalent to 8.9 ICU beds per 100 000 population, more than the UK but fewer than Italy and the US.^5^, ^6^

In late March 2020, rising numbers of COVID‐19‐related admissions to ICUs were observed throughout Australia.^7^ The Australian and New Zealand Intensive Care Society (ANZICS) and the Australian Government Department of Health recognised that ICU demand was unlikely to be uniform, that capacity might be exceeded in one region but not in another, and that matching ICU resources to areas of greatest need might be required. A single sentence encapsulated the approach: “Why would we let a patient die in Western Australia if we can see a spare ventilator in Sydney?”

## A nationwide system to monitor ICU demand and capacity in Australia

A nationwide dashboard of ICU activity, the Critical Health Resources Information System (CHRIS), was rapidly developed as a collaboration between Telstra Purple, Ambulance Victoria, ANZICS and the Australian Government Department of Health. All adult and paediatric ICUs (public and private) in Australia were instructed to enter data twice daily. This manual data entry typically took 5 minutes. Each ICU was immediately able to see patient numbers and resources available within every ICU in their region and also see an aggregate summary of all ICUs in Australia. CHRIS was available to all state and territory health departments, to all patient transport and retrieval agencies, and also to ICUs in New Zealand. The system went live on 1 May 2020, after 26 days of development. Three weeks later, 184 out of 188 eligible ICUs (98%) in Australia were contributing data.

## The ICU response to the second wave of COVID‐19 in Victoria

After a decline in severe acute respiratory syndrome coronavirus 2 (SARS‐CoV‐2) infections throughout Australia, notifications rose again in Melbourne at the end of June 2020.^8^ In response, ICU directors from the lead hospitals of the nine designated Victorian health care clusters commenced a daily morning meeting with representatives from Ambulance Victoria, Safer Care Victoria and the Victorian Department of Health and Human Services. The group committed to maintaining standards of care expected under normal (non‐pandemic) conditions and to achieving this by proactively transferring patients (with or without COVID‐19) to another ICU if delivery of care was compromised by high local demand. Decisions to transfer patients were informed by data from CHRIS. Pre‐existing critical care transfer systems run by Ambulance Victoria were used.

From the beginning of July to the end of September 2020, there were 237 ICU admissions with COVID‐19 pneumonitis, of which 210 (88%) occurred in July and August. Admissions were predominantly to public hospitals in north‐western Melbourne.^9^ The rapid and localised nature of presentations meant that it was faster to transfer patients to ICUs with vacant capacity than to open and staff additional beds, despite physical ICU bed spaces being available. Transfers from the emergency department or ICU at the four north‐western metropolitan hospitals alone accounted for 35% (46/133) of all critical care transfers in Victoria during July and August.

Spare ventilators were available at all sites on all days. On six occasions in August, there were more than 140 ventilated patients (with or without COVID‐19) in Victoria. On each of these days, there were more than 500 spare ICU ventilators available (Box 1 and Supporting Information, graphic 1 in the video). Despite individual hospitals indicating transient increases in ICU bed numbers, there was no overall increase in open staffed ICU beds. As COVID‐19 cases rose, so too did numbers of critical care staff unavailable due to COVID‐19 exposure or illness, with 15 consecutive days when there were more than 60 staff unavailable (Box 2).

Box 1 **Snapshot of the Critical Health Resources Information System (CHRIS) summary page for Victoria during August 2020**

**Figure:**
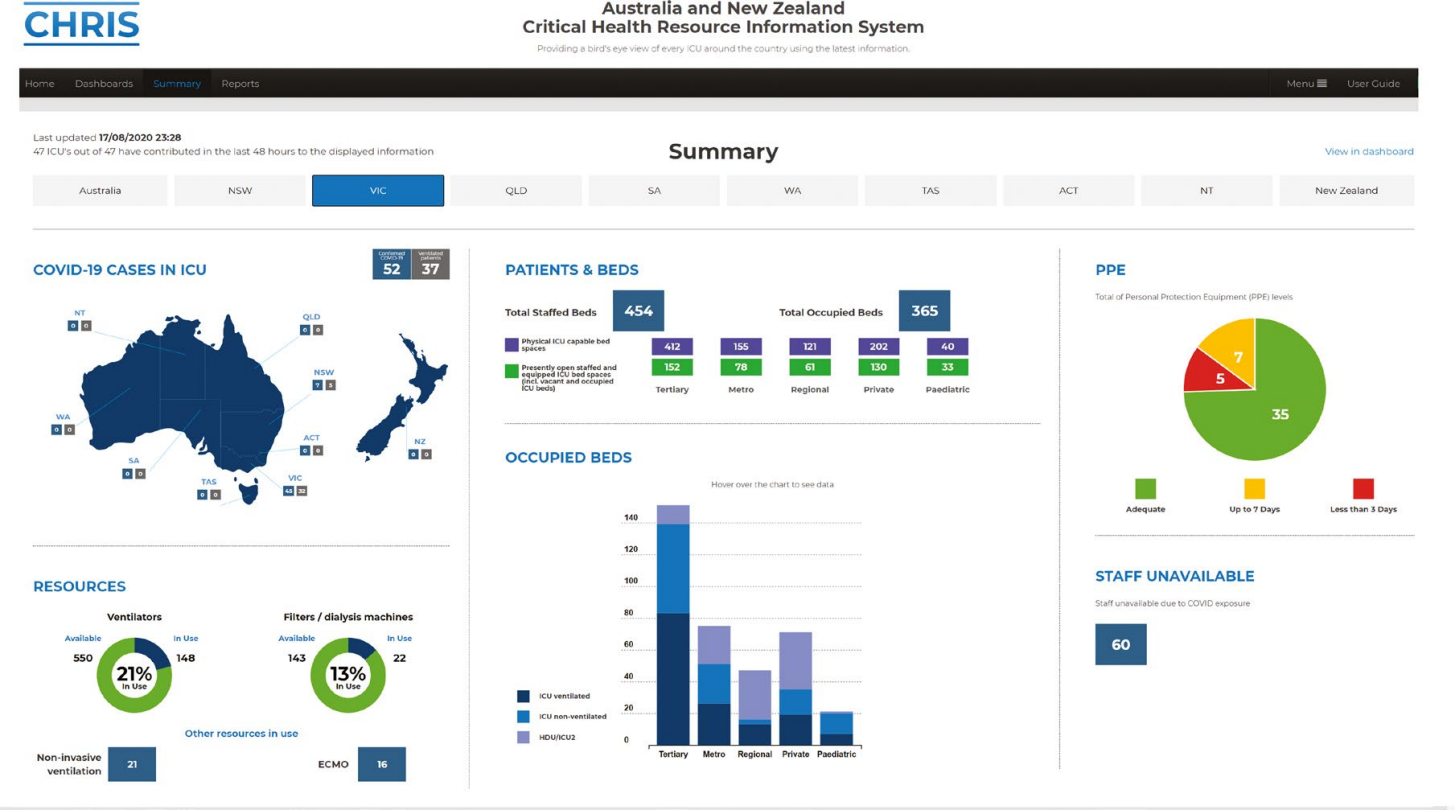
ACT = Australian Capital Territory; COVID‐19 = coronavirus disease 2019; ECMO = extracorporeal membrane oxygenation; HDU = high dependency unit; ICU = intensive care unit; NSW = New South Wales; NT = Northern Territory; NZ = New Zealand; QLD = Queensland; SA = South Australia; TAS = Tasmania; VIC = Victoria; WA = Western Australia.

Box 2 **Number of ventilated (dark blue) and non‐ventilated (light blue) patients in Victorian intensive care units and the number of critical care staff unavailable to work due to coronavirus disease 2019 (COVID‐19) exposure or illness (green dots), listed each morning in the Critical Health Resources Information System (CHRIS)**

**Figure:**
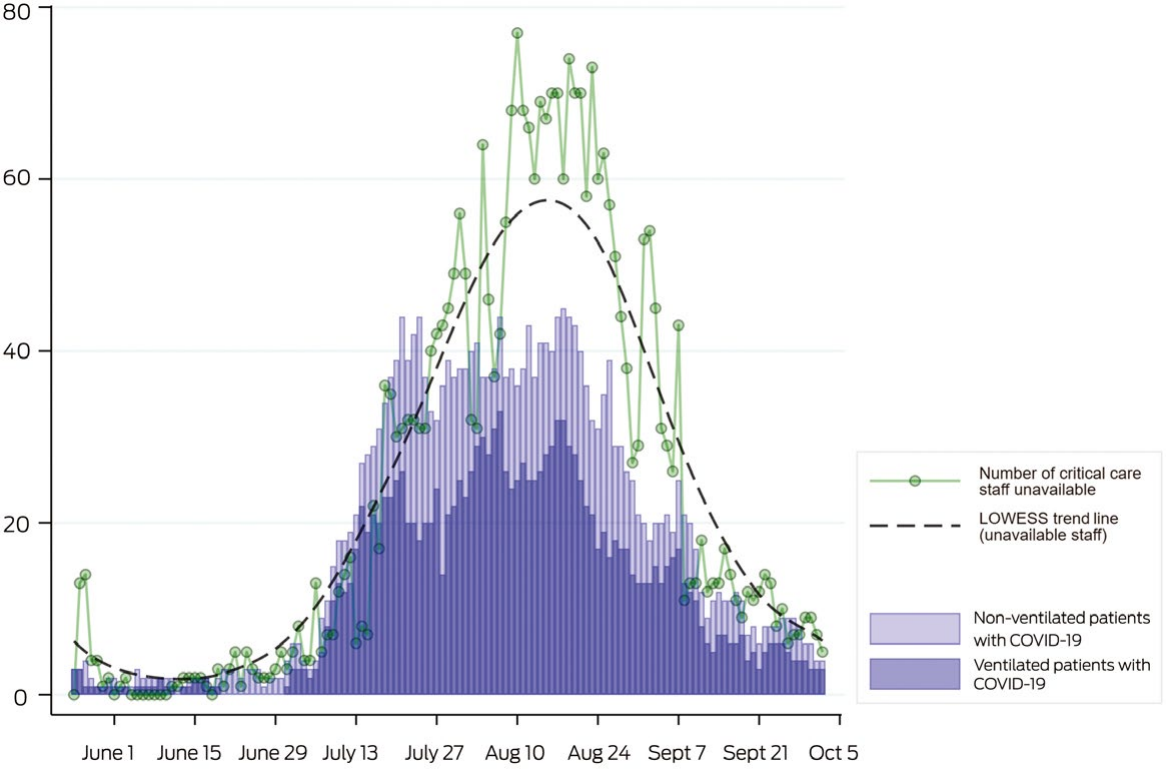
LOWESS = locally weighted scatterplot smoothing.

## Lessons learned

CHRIS provided real‐time data on ICU activity and capacity. In addition to facilitating the transfer of critically ill patients, CHRIS also enabled early diversion of ambulance presentations to emergency departments at hospitals where ICUs had capacity. These approaches were integral to ensuring standards of care were maintained by clinicians, retrieval agencies and the Victorian health department. At the same time, there was visibility to the Australian Government Department of Health, which would, if required, coordinate a national response to overwhelmed ICU services.

Although several individual ICUs came under strain, retrieval and critical care systems in metropolitan Melbourne were not overwhelmed. Strategies to redistribute critical care demand are likely to have contributed to high survival rates for ventilated patients with COVID‐19 in Victoria.^9^ Timely transfers to ICUs with open available beds could be facilitated. Availability of staff was more important in determining capacity to deliver care than availability of ventilators.

## The role for CHRIS in the future

The local application of a national tool (CHRIS) for real‐time display of ICU activity and resources was a key component of the response to the COVID‐19 pandemic in Victoria. CHRIS has the potential to augment existing ICU monitoring systems. The tool may also assist in the response to local and national public health emergencies, such as mass casualty events, bushfires^10^ or thunderstorm asthma.^11^ Automated linkage of CHRIS to existing state‐based and national systems should be investigated. In addition, it may have potential use in monitoring health policy impacts more broadly.

## Competing interests

No relevant disclosures.

## Provenance

Not commissioned; externally peer reviewed.

## Supporting information

Video S1

## References

[1] Yang X , Yu Y , Xu J , et al. Clinical course and outcomes of critically ill patients with SARS‐CoV-2 pneumonia in Wuhan, China: a single‐centered, retrospective, observational study. Lancet Respir Med 2020; 8: 475–481. 32105632 10.1016/S2213-2600(20)30079-5 PMC7102538

[2] Grasselli G , Pesenti A , Cecconi M . Critical care utilization for the COVID‐19 outbreak in Lombardy, Italy: early experience and forecast during an emergency response. JAMA 2020; 323: 1545–1546. 32167538 10.1001/jama.2020.4031

[3] Richardson S , Hirsch JS , Narasimhan M , et al. Presenting characteristics, comorbidities, and outcomes among 5700 patients hospitalized with COVID‐19 in the New York City area. JAMA 2020; 323: 2052–2059. 32320003 10.1001/jama.2020.6775 PMC7177629

[4] Litton E , Bucci T , Chavan S , et al. Surge capacity of intensive care units in case of acute increase in demand caused by COVID‐19 in Australia. Med J Aust 2020; 212: 463–467. https://www.mja.com.au/journal/2020/212/10/surge-capacity-intensive-care-units-case-acute-increase-demand-caused-covid-19 32306408 10.5694/mja2.50596 PMC7264562

[5] Prin M , Wunsch H . International comparisons of intensive care: informing outcomes and improving standards. Curr Opin Crit Care 2012; 18: 700–706. 22954664 10.1097/MCC.0b013e32835914d5 PMC3551445

[6] Rhodes A , Ferdinande P , Flaatten H , et al. The variability of critical care bed numbers in Europe. Intensive Care Med 2012; 38: 1647–1653. 22777516 10.1007/s00134-012-2627-8

[7] Burrell AJC , Pellegrini B , Salimi F , et al. Outcomes for patients with COVID‐19 admitted to Australian intensive care units during the first four months of the pandemic. Med J Aust 2021; 214: 23–30. https://www.mja.com.au/journal/2021/214/1/outcomes-patients-covid-19-admitted-australian-intensive-care-units-during-first 33325070 10.5694/mja2.50883

[8] O’Reilly GM , Mitchell RD , Mitra B , et al. Epidemiology and clinical features of emergency department patients with suspected and confirmed COVID‐19: a multisite report from the COVED Quality Improvement Project for July 2020 (COVED‐3). Emerg Med Australas 2021; 33: 114–124. 32959497 10.1111/1742-6723.13651 PMC7536936

[9] Australian and New Zealand Intensive Care Society Centre for Outcome and Resource Evaluation . Report on COVID‐19 admissions to intensive care in Victoria (01 January to 30 September 2020). Melbourne: ANZICS, 2020. https://www.anzics.com.au/wp-content/uploads/2020/10/ANZICS-CORE-COVID-19-Report_VIC_01-Jan_30-Sept.pdf (viewed Oct 2020).

[10] Vardoulakis S , Jalaludin BB , Morgan GG , et al. Bushfire smoke: urgent need for a national health protection strategy. Med J Aust 2020; 212: 349–353. https://www.mja.com.au/journal/2020/212/8/bushfire-smoke-urgent-need-national-health-protection-strategy 32088929 10.5694/mja2.50511 PMC7318141

[11] Darvall JN , Durie M , Pilcher D , et al. Intensive care implications of epidemic thunderstorm asthma. Crit Care Resusc 2018; 20: 294–303. 30482137

